# T cells: an emerging cast of roles in bipolar disorder

**DOI:** 10.1038/s41398-023-02445-y

**Published:** 2023-05-08

**Authors:** Zhenni Chen, Yiran Huang, Bingqi Wang, Huanqie Peng, Xiaofan Wang, Hongzheng Wu, Wanxin Chen, Min Wang

**Affiliations:** 1 Department of Laboratory Medicine, the Second Xiangya Hospital of Central South University, Changsha, Hunan 410011 China; 2 School of Clinical Medicine, Hunan University of Chinese Medicine, Changsha, Hunan 410208 China

**Keywords:** Bipolar disorder, Physiology

## Abstract

Bipolar disorder (BD) is a distinctly heterogeneous and multifactorial disorder with a high individual and social burden. Immune pathway dysregulation is an important pathophysiological feature of BD. Recent studies have suggested a potential role for T lymphocytes in the pathogenesis of BD. Therefore, greater insight into T lymphocytes’ functioning in patients with BD is essential. In this narrative review, we describe the presence of an imbalance in the ratio and altered function of T lymphocyte subsets in BD patients, mainly in T helper (Th) 1, Th2, Th17 cells and regulatory T cells, and alterations in hormones, intracellular signaling, and microbiomes may be potential causes. Abnormal T cell presence explains the elevated rates of comorbid inflammatory illnesses in the BD population. We also update the findings on T cell-targeting drugs as potentially immunomodulatory therapeutic agents for BD disease in addition to classical mood stabilizers (lithium, valproic acid). In conclusion, an imbalance in T lymphocyte subpopulation ratios and altered function may be involved in the development of BD, and maintaining T cell immune homeostasis may provide an overall therapeutic benefit.

## Introduction

Bipolar disorder (BD) is a chronic disease characterized by episodes of major depression and mania or hypomania that affect >1% of the global population [1, 2]. Around two-thirds of patients with BD also experience severe emotional and cognitive impairments and difficulty handling daily activities [2]. The effective treatment and prevention of BD represent an area of significant unmet medical need, it remains vital to understand the pathophysiological mechanism of BD for clinical management. Multiple factors, such as genetic, environmental, biological, and psychological, are involved in the pathogenesis of BD [3]. As an essential component in maintaining the homeostasis of the internal environment, the immune system has typically evolved to protect humans from infection and malignancy; nonetheless, it is not without effects on the central nervous system, which can lead to changes in mood, cognition, and behavior [4]. The role of immune dysfunction in BD is currently unclear, with low-grade chronic inflammation (increased plasma cytokines, soluble cytokine receptors, chemokines, acute phase reactants) and T-cell activation features that may be associated with BD, but the results are controversial [5–9]. Stressful situations can trigger emotional episodes in individuals with BD, and T cells are associated with the physiological stress response [10, 11]. Different subsets of T cells with highly diverse functions can be distinguished based on CD protein expression, chemokine receptor expression, and the cytokines they produce. T cells can be classified as helper or cytotoxic depending on whether they are CD4^+^ or CD8^+^ respectively [12]. Naïve T helper cells can differentiate into various subtypes which can further complicate their role in stress susceptibility and resilience [11]. Several studies have suggested that there are changes in the number and function of T-lymphocyte subsets in BD patients [13, 14]. To date, how T lymphocytes influence the onset and progression of BD and how these processes are regulated is still unknown. The participation of T lymphocytes and their cytokines in BD will be reviewed, focusing on controversial and recent findings, including the relative abundance and altered function of different subpopulations. The potential causes of this phenomenon are explored. Finally, alterations in T lymphocytes in BD are associated with long-term negative outcomes and can influence disease outcomes through immune regulation. Together, this review will highlight the contribution of T cells to BD pathology and offer a future perspective on a potential translation into clinical practice by targeting T cells to modulate BD.

## T lymphocytes and BD

In the absence of active somatic immune diseases, abnormalities of T cell-mediated immunity have been reported in BD [13]. Progressively increased vulnerability to developing inflammatory and compromised lymphoproliferative response correlates with the severity of disease in BD patients [15]. Alterations in T lymphocytes appeared during the exacerbation of BD and correlated significantly with the results of the Hamilton Rating Scale for depression [16] or Young Rating Scale for Mania [17], i.e., the higher the patient’s score (the higher the degree of depression or mania), the lower the percentage of T lymphocytes, which tended to normalize after successful treatment/remission [14]. BD patients also display early T-cell senescence linked with premature aging and neuro-progression [18]. Some studies have claimed a decrease in circulating T cells [14, 19], and others have shown that total T cells are significantly higher in patients with BD than in healthy controls (HC) [20, 21], or that there is no change [22]. These conflicting reports may be due to differences in the severity and duration of the disease, or the fact that patients are receiving mood-stabilizing agents (e.g., lithium, anticonvulsants, and antipsychotics) that may influence immune function or the analysis of different T-cell subsets. To date, the exact changes in T lymphocytes are controversial (Table 1), but there is a proven correlation between chronic activation of the immune system response by T lymphocytes and mood dysregulation [23].

**Table 1:** Summary of studies of T-lymphocyte changes in BD patients.

Study	Patients	Controls	Markers	Immune subsets/Identifies	Significant T-cell-related findings
Wahlin A et al., 1984 [121]	37	12	CD4, CD8	Th, Tc	Patients with BD had no difference in T lymphocyte subsets compared to HC in the pre-lithium treatment period, and after lithium treatment, Th cells decreased and Tc cells increased.
Wilson R et al., 1991 [133]	40	40	CD3, CD4, CD8	Th, Tc	Peripheral blood mononuclear cells from patients receiving lithium were found to have significantly reduced numbers of suppressor/cytotoxic T cells.
Breunis MN et al., 2003 [20]	64	38	CD3, CD4, CD8, CD25, CD69, CD71, MHC II	Th, Tc, activated T cells	Significantly higher numbers of circulating activated T cells were found in BD patients compared with HC.
Torres KC et al., 2008 [134]	7	7	CD3, CD4, CD28	Th	The higher percentage of CD4^+^ cells in BD patients than in controls.
Drexhage RC et al., 2011 [13]	38	22	CD3, CD4, CD25, FOXP3, IL-4, IFN-γ, IL-17A	Th, Th1, Th2, TH17, Tregs	Compared with HC, the percentages of anti-inflammatory Tregs were higher, and the percentages of Th1, Th2, and Th17 cells were normal in BD.
Wieck A et al., 2013 [40]	13	15	CD3, CD4, CD8, CD25, CD28, CD45RO, CD45RA, FOXP3, CCR7	Th, Tc, Th1、Th2、Th17, Tregs	Compared to HC, BD patients had an increased percentage of activated T cells and decreased Tregs, memory T CD8^+^ cells, and naïve T CD8^+^ cells.
do Prado CH et al., 2013 [41]	27	24	CD3, CD4, CD8, CD25, CD28, CD45RO, CD45RA, FOXP3, CCR7	Th, Tc, Th1、Th2、Th17, Tregs	Compared to HC, BD patients had a reduced proportion of Tregs and increased senescence-associated cells (CD8^+^ CD28^-^), associated with a strong bias towards Th1, rather than a Th2 profile.
Barbosa IG et al., 2014 [19]	21	21	CD3, CD4, CD8, CD25, FOXP3, IL-10	Th, Tc, Tregs	BD patients presented reduced proportions of total T cells and Tc. BD patients also exhibited a higher percentage of activated T cells and a lower percentage of IL10-expressing Tregs.
Wu W et al., 2017 [29]	23	20	CD3, CD4, CD8	Th, Tc	The blood proportion of Tc cells significantly decreased in BD patients than in HC.
Vogels RJ et al., 2017 [33]	97	72	CD4, CD8, CD25, IFN-γ, IL-4, IL-17A, FOXP3	Th, Tc, Th1, Th2, Th17, Tregs	Total T cells were reduced in BD patients, and among them, there were changes in the Th cell population, with a significant increase in the percentage of Th2 and Th17 cells and a decrease in Tregs.
Poletti S et al., 2017 [47]	25	21	CD3, CD4, CD25, CXCR3, CCR4, CCR6, CCR10, CD161, FOXP3	Th, Th1, Th2, Th17, Th22, Tregs	The percentage of circulating Th17 cells correlated positively with higher fractional anisotropy in fiber tracts contributing to the functional integrity of the brain both in BD patients and HC, while the frequency of circulating Tregs correlated positively with higher radial and mean diffusivity in BD patients.
Becking K et al., 2018 [22]	91	165	CD3, CD4, CD8, CD25, IL-4, IFN-γ, IL-17A, FOXP3	Th, Tc, Th1, Th2, Th17, Tregs	In BD patients, levels of Th17 and Tregs were increased compared to HC.
Magioncalda P et al. 2018 [65]	60	20	CD3, CD4, CD8, CD28, CD45RA, IFN-γ	Th, Tc, CD8^+^CD28^-^ T cell subsets	White matter abnormalities are highly correlated with a reduction in circulating CD8^+^ T cell subpopulations that are terminally differentiated effector cells prone to tissue migration.
Counotte J et al., 2018 [38]	87	46	CD3, CD4, CD8, CD45RO, CD25, IFN-γ, IL-4, IL-17A, FOXP3	Th, Tc, Th1, Th2, Th17, Tregs	In the high psychosis liability group, childhood trauma was associated with increased Th17, with increased Tregs predicted stress experienced during exposure to virtual social stressors.
Snijders G et al., 2019 [135]	52	54	CD3, CD4, CD8, CD25, FOXP3, IL-4, IFN-γ, IL17A	Th, Tc, Th1, Th2, Th17, Tregs	The mean levels of T cell percentage in the index and co-twins were significantly lower compared to the HC twins. T-cell percentages were not significantly different in the group of BD patients with an active mood episode versus those in a euthymic state.
Lu Q et al., 2019 [86]	36	27	CD3, CD4, CD8	Th, Tc	At baseline, the CD3^+^ T cell proportion was positively correlated with log_10_ *Enterobacter spp* count, whereas the correlativity between the other bacteria and immune profiles was negative.
Wu TN et al., 2019 [21]	76	60	CD3, CD4, CD8, CD25, CD28	Th, Tc, Tregs, activated T cells	BD patients had significantly higher percentages of total T and Th cells than HC.
Pietruczuk K et al., 2019 [14]	22	14	CD3, CD4, CD8, CD25	Activated T cells	The percentage of activated T cells was decreased in patients in the hypomanic phase compared with HC.
Maes M et al., 2021 [15]	25	21	CD3, CD4, CD8, CD71, CD69, CD25, CD152, CD154, FOXP3	Activated T effector, Tregs	BD and anti-HCMV IgG levels significantly interact to decrease the expression of CD4^+^ CD25^+^ FOXP3^+^ T phenotypes.
Su L et al., 2022 [136]	83	101	CD3, CD4, CD8, CD45	Th, Tc	BD patients had greater CD3^+^ T cell levels than HC.

The inclusion and exclusion criteria for the studies are shown in Supplementary Fig. 1. The quality assessment is shown in Supplementary Table 1.

*HC* healthy controls, *Th* T helper, *Tc* cytotoxic T cell, *Tregs* regulatory T cells, *IL* interleukin, *IFN* interferon, *FOXP* fork-head box protein, *CXCR* CXC chemokine receptors, *CCR* chemokine receptor, *MHC* major histocompatibility complex, *HCMV* human cytomegalovirus.

In mouse models, chronic stress reduces immune responses, including reduced leukocyte trafficking, neutrophil phagocytosis, and reduction of peripheral lymphocytes [24]. Severe combined immune deficiency mice exhibit cognitive impairments that can be reversed by restoring a proper immune response. T cell immunodeficient mice show a reduced ability to deter the consequences of stress and had raised anxiety responses; a short exposure of mice to the predator odor stressor enhanced T cell infiltration into the brain via the choroid plexus, and the ability of mice to cope with the stress was interrelated with the T cell trafficking [25]. The depressive and anxiety-like behavior exhibited by *Rag1*^*–/–*^ or *Rag2*^*–/–*^ mice lacking T cells can be improved by transplantation of splenocytes/lymphocytes from chronically stressed mice, and immunization with myelin-related peptides can further reduce stress-induced anxiety [26–28]. Animal results further confirm that T-cell immune homeostasis can suppress excessive behavior and anxiety responses induced by stressful stimuli. Indeed, as immune cell immunophenotyping studies progressed, T cell subsets associated with BD were explored in depth, and specific subtype variations are discussed in more detail in the next section.

### T-cell subsets and BD

#### CD4^+^ T subsets and BD

**Fig. 1:**
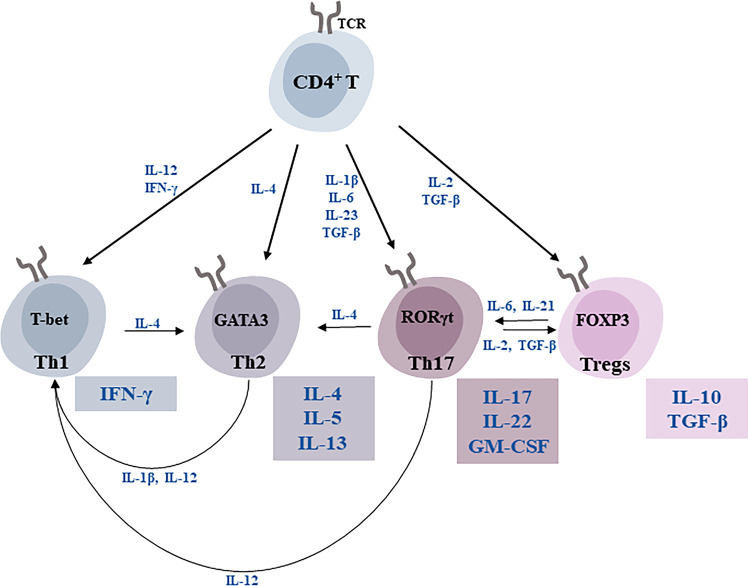
CD4^+^ T cells and cytokines implicated in BD. Once activated by their antigen and the cytokine milieu (each lineage of CD4^+^ T cells has a combination of cytokines required to differentiate), as well as upon receiving co-stimulatory signals, CD4^+^ T cells differentiate in the different lineages: Th1, Th2, Th17 cells, and Tregs, which produce a combination of cytokines as indicated. These effector T cells are considerable plasticity allowing for conversion to other phenotypes [137]. TCR T cell receptor, T-bet T-box transcription factor, RORγt retinoic acid related orphan receptor γ t, GM-CSF granulocyte-macrophage colony-stimulating factor.

In response to the milieu, CD4^+^ T cells differentiate into a specific lineage belonging to a continuum of phenotypes (Fig. 1). The main lineages studied in BD are: Th1, Th2, Th17 cells, and Regulatory T cells (Tregs), the alterations in cytokines may be due to changes in T-cell subsets. A preliminary study aimed to investigate the activation pattern of T lymphocyte populations in BD patients, the blood cell counts of T-lymphocyte subsets, and the plasma levels of cytokines they secreted were selectively investigated. As a result, the plasma level of IL-6 was found to be elevated in patients with BD [29]. Changes in cytokines in BD patients of different features varied in degree and were related to the course of the disease and the characteristics of symptoms and may alter after medication [30]. CD4^+^ T cells demonstrate a shift from the Th1-phenotype toward others during natural senescence, which could be exaggerated by BD [31]. The Th1 chemokine receptor-5 is downregulated in BD, and serum levels of Interferon-γ (IFN-γ), a cytokine produced by Th1, are significantly reduced, and in vitro stimulation of lymphocytes also results in reduced IFN-γ release [19, 32], while Th2 levels elevated and its marker IL-4 is also upregulated in BD [33, 34]. These suggest a Th1/Th2 shift in BD. Studies have confirmed that pharmacological treatment can restore the down-regulated Th1/Th2 [35]. In addition, Th17 are pro-inflammatory neurotoxic cells, whereas Tregs are anti-inflammatory neuroprotective cells. The balance between these two subsets is essential for immune homeostasis, and dysregulation of this balance has been implicated in various inflammatory conditions, allograft rejection, and tumorigenesis [36]. Compared to HC, the levels of both Th17 cells and their cytokine IL-17 production were significantly increased in BD patients and correlated positively with the duration of the disease [22, 37]. In the high psychiatric risk group, childhood trauma was associated with increased Th17 cell numbers, but it has also been shown that biphasic offspring have reduced Th17 cells in early adulthood and that there may be a correlation between altered Th17 and age [38, 39]. Tregs prevent immune responses against self-antigens and reduced Tregs and secreted IL-10 in BD could explain the increased incidence of the autoimmune disease reported in individuals [19, 40–43]. Adolescent offspring of BD patients also show a subtle reduction in Treg numbers and a negative correlation with inflammatory monocyte activity [39]. It has also been found that there are elevated levels of Tregs in BD patients or that there are no significant differences with HC [13, 22]. The sources of immuno-inflammatory activation in BD are multifactorial (eg, epigenetic modification, intrinsic physiologic alterations in BD, and exposure to social adversity during early life) [44]. The BD population may have different immune biotypes or be at different stages of the disease, leading to differences in clinical findings [44, 45]. Thus, the causal relationship between immune activation brought about by CD4^+^ T cells and altered emotional reactivities required further confirmation.

Th and Tregs are essential for normal brain development and when their function is impaired may affect brain function in BD patients [46]. Poletti et al. focused on the relationship between circulating levels of Th and Tregs with structural and functional brain imaging in BD patients and showed consistency between Th and Tregs cell-mediated immune activation maintains the functional and structural integrity of the brain [47]. The balance between the Th17 and Tregs might regulate the White matter (WM) microstructure of key tracts in interhemispheric, prefrontal-occipital, and temporal-frontal-occipital connections that are critical for cognitive and emotional brain function [47]. In addition, there is controversy about the site of action of these Th and Tregs in brain development and function. Some consider the activity of the cells in the peripheral lymphoid organs as sufficient to induce these brain effects (with T cell-specific cytokines entering the brain via the circulation) [46], while others assume that the T cells themselves traffic to the brain to exert their effects [48]. Indeed, there is a special CC-chemokine ligand 20/CC-chemokine receptor 6 dependent route for Th17 cells and Tregs to enter the cerebrospinal fluid via the choroid plexus, delivering the cells in close vicinity to the limbic structures [49, 50]. During the inflammatory process, the release of cytokines may induce permeability of the blood-brain barrier, thus allowing peripheral pro-inflammatory mediators to enter the brain, constituting a bidirectional transfer of inflammatory substances [51]. Elevated levels of pro-inflammatory cytokines impact synaptic transmission and negatively affect neuronal survival and may precipitate learning and memory impairment [52]. The upregulation of immune response genes in the prefrontal and orbitofrontal cortex of BD patients confirms these findings and reveals the connection between changes in peripheral inflammatory markers and neuroinflammation in the pathophysiology of BD [53, 54]. Brain-derived neurotrophic factor (BDNF) is a neurotrophic producer that is thought to play an essential role in neuro-regeneration and neuroplasticity and to provide the necessary conditions for normal nerve cell function, and impaired CD4^+^ T-cell activity results in reduced expression of BDNF in the brain [46]. Current evidence indicates decreased BDNF levels in BD patients compared to HC and correlations between BDNF and the severity of symptoms/mood episodes [55]. The above results suggest that the immune system and neurotrophic signaling interact to promote disease activity and neurological progression in BD.

Chronic stress-induced mice models have mitochondrial metabolic dysfunction in CD4^+^ T cells [56]. Lymphocyte subsets adoptively transferred into or selectively deleted from mice can exert effects on cognition [26, 56–58]. Rattazzi argued that CD4^+^ T cells correct a naturally existing anxious phenotype in lymphocytopenia *Rag1*^*–/–*^ mice [26]. In contrast, the transfer of CD4^+^ T cells from naive mice to *Rag1*^*–/–*^ mice did not affect anxiety behavior [57]. Fan confirmed that naïve CD4^+^ cells but not effector T cells from electric foot shock-treated donor mice adequately conferred behavioral abnormalities [56]. Besides, Kim showed that depleting Tregs caused an increase in anxiety-like behavior in the elevated plus maze [58]. Remarkably, not only were the behavioral decline and decreased cell proliferation associated with stress reversed in the mice receiving cells from stressed donors, but the pro/anti-inflammatory blood cytokine profile was also substantially reversed between donor and host [57]. *Integrin β4*^*–/*–^ (*ITGB4*^*-/-*^) mice have BD-related cognitive dysfunction, and expression levels of the pro-inflammatory cytokines TNF-α and IL-6 were higher in the hippocampus and prefrontal cortex of *ITGB4*^*–/–*^ mice than in control mice [59]. *ITGB4*^*–/–*^ mice mainly exerted Th2-type inflammation in the periphery, like the number and major cytokines IL-4 and IL-13 of Th2-type inflammation [59]. By binding the IL-4 receptor alpha chain in the choroid plexus, IL-4 induces the release of pro-inflammatory cytokines from macrophages, which then leakage into the brain and promote the production of a second wave of cytokines by microglia, leading to neural network dysfunction, which then triggers the onset of BD [59, 60]. Studies in rodents provide corroborative further evidence of the involvement of CD4^+^ T cells in BD.

High levels of Th1 and Th17 are generally considered a sign of inflammation, while high levels of Th2 and Tregs are anti-inflammatory. Mainstream findings suggest that there is upregulation of Th2 and Th17 and downregulation of Th1 and Tregs in BD patients, suggesting activation of both pro- and anti-inflammatory forces. Similar combinations of increases and decreases of pro- and anti-inflammatory forces have been described in BD and other psychiatric disorders [61, 62]. This suggests not so much a pro- or anti-inflammatory state as the hallmark of psychiatric disease with an exaggeration or deficiency of immune activity. The balanced increases and decreases of Th and Tregs in BD patients, as described here, suggest another role for CD4^+^ T cells in regulating neural activity, not just inflammatory regulation.

#### CD8^+^ T cells and BD

Cytotoxic T cells (CD8^+^ cells) are involved in antiviral, cancer, reactive rejection, and immunomodulatory cellular immunity by killing infected cells. The co-stimulatory molecule CD28 is necessary to initiate T-cell-mediated immune responses. The dramatic loss of telomerase activity in CD8^+^ CD28^-^ T cells limit their proliferation ability [63], while the reduced telomere length in lymphocytes of BD patients is associated with the number of lifetime depressive episodes, suggesting that early aging of the immune system may be related to the pathophysiology of BD [64]. Aging is commonly associated with the progressive loss of the CD28 marker and increased CD8^+^ CD28^-^ T cell subpopulations in the blood [63]. do Prado et al. further found an expansion of senescence-associated cells (CD8^+^ CD28^-^ T cells) in BD, but the results are controversial [41]. It has also been found that the percentage of CD8^+^ T cells in BD patients is reduced both during disease onset and remission, with the reduced CD8^+^ T cells belonging to the CD8^+^CD28^-^ and IFN-γ^+^ subpopulations (corresponding to functionally activated CD8^+^ T cells) [14, 19, 40, 65]. The reduced percentage of CD8^+^ T cells in BD patients supports the hypothesis that there is a close relationship between BD and virus-induced infections. Also, the study proposes that CD8^+^ T cells in the blood of BD patients are chronically immune activated, leave the circulating blood, and migrate to peripheral tissues, including brain regions, and that reduced WM integrity in the corpus callosum and left supra-radial corona is associated with reduced end CD8^+^ CD28^-^ and CD8^+^ IFN-γ^+^ populations [65]. This phenomenon is reminiscent of chronic inflammatory neurological diseases such as multiple sclerosis. There, a reduction of CD8^+^ T cells and increased CD4^+^/CD8^+^ ratio in the peripheral blood circulation correlated with the accumulation of CD8^+^ T cells in acute and chronic inflammatory WM lesions are observed, suggesting that CD8^+^ T cells recognize myelin components and could act as effector cells to promote WM injury [66]. Future studies will need to formally demonstrate the presence and localization of specific subpopulations of CD8^+^ T effector cells in the post-mortem brain of BD subjects to prove their pathogenic potential in BD.

### Possible origins of the T-cell dysregulations in BD

Although it remains to be determined whether all the dysregulation of T cells observed in patients with BD occurs simultaneously in the same individual, it is conceivable that BD-specific factors may affect T cell function. Thus, for example, the plasticity of Th cells may be responsible for some of the differences observed between different Th subtypes in BD. It is also possible that previous treatments with mood stabilizers affect the proportion of Th cells. In addition, mood dysregulation may be directly linked to stressors, and T cells may be affected by different stressors to produce different changes, including the source of stress (internal versus external), the stress hormones, as well as indirect effects of stress, such as alterations of the microbiota, which is known to control immunity, and some of the pathways leading to these different stress behaviors are described below.

#### Hormones

Exposure to stress leads primarily to activation of the Hypothalamic-pituitary-adrenal (HPA) axis and the sympathetic nervous system to regulate metabolism, neuronal and cardiovascular functions, as well as immune activity. The hippocampus, amygdala, and prefrontal cortex undergo stress-induced structural remodeling, which alters behavioral and physiological responses [67]. Cortisol, the main by-product of the HPA axis, has both adaptive and maladaptive effects on these brain regions, which could influence cognitive and emotional functioning [68]. With chronic inflammation, HPA activation may cause harmful effects by chronic hypercortisolemia, which downregulates the synthesis of Glucocorticoids (GCs) receptors, translocation, and sensitivity in the pituitary and hypothalamus and effectively inhibits the negative feedback loop of the HPA axis in BD [69]. The negative feedback loop leads to further impairments in mood and cognition with increasingly elevated cortisol levels. GCs abnormalities are present in BD patients, and higher cortisol levels are positively correlated with manic phases and negatively correlated with the use of antipsychotic medication [70]. Endogenous GCs are involved in the selection of appropriate T cell receptor pools in the thymus, suppressing Th1 cells while moderately suppressing Th2 cells and favoring Th17 cells, regulating T cell trafficking and promoting T cell memory; exogenous GCs (e.g., corticosteroid treatment) form cytokines required for T cell differentiation by blocking gene expression of a variety of cytokines, including IL-1, IL-2, IL-3, IL-4, IL-5, IL-6, IL-8, IL-10, IL-13, GM-CSF, TNF and IFN-γ environment [71]. Glucocorticoid receptors are expressed on all T cells, but different T cells have different sensitivity to glucocorticoids. The difference is mainly attributed to the glucocorticoid-induced loss of expression of B-cell lymphoma 2 (BCL-2), and greater expression of BCL-2 in Th1 cells, whereas BCL-2 is moderately affected in Th2 cells and increased in Th17 cells [72]. Furthermore, Tregs differentiation is also promoted by GCs due to the upregulation of TGF-β receptors and Foxp3, and the promotion of Tregs by GCs might be one of the major mechanisms mediating immunosuppression [73]. The widespread immune effects of GCs may explain the enrichment of certain T cell subtypes during BD, e.g., Th17 cells are the most resistant cells to glucocorticoid-induced apoptosis and are found elevated in BD, but the suppressive effect of GCs on Th2 cannot explain the elevation of Th2 in BD patients, so other factors combine to promote the abnormal immune-inflammatory process in BD. The catecholamines include neurotransmitters such as epinephrine, norepinephrine, and dopamine and act through the autonomic nervous system. Traditionally, the sympathetic nervous system is activated after stress to increase blood epinephrine and norepinephrine levels. Immune cells express both α-adrenergic and β-adrenergic receptors, and their level of expression depends on the state of the cells, their maturation, and their activation [74]. Consistent with this, norepinephrine promotes the transfer of Th1 cells to Th2 cells, a phenomenon that explains the Th1/Th2 shift existing in BD patients [75]. There is a balance between epinephrine promoting Th17 cells through secretion of cytokines by the dendritic cells [76] and norepinephrine directly inhibiting Th17 cells through β2-adrenergic receptors [77]. Additionally, sympathetic dopaminergic innervations are present in the thymus, spleen, and lymph nodes. Both peripheral and CNS immune cells express all dopamine receptors, transporter proteins, and related enzymes; dopamine receptors hypofunction is associated with altered Tregs and impaired transport of immune cells to the brain [24]. Nevertheless, the duration and concentration of exposure to the catecholamines, the interaction with other factors induced during stress (GCs, etc.), and the role of adrenergic receptors in the various T cell subtypes need to be also taken into consideration. Other nervous systems also affect T cells, including the parasympathetic nervous system with the release of acetylcholine and the sensory nervous system with the release of neuropeptides and glutamate, which are also known to modulate T cell responses [78].

#### Intracellular signaling

Many BD patients experience a chronic, low-grade inflammatory state that may be enhanced to varying degrees during acute emotional episodes, which may exacerbate the T-cell deficits noted in this review and contribute to overall cognitive decline and neurological progression. Inflammatory signaling mediates a strong connection between cellular stress, neuronal viability, and apparent symptoms, and differences in intracellular signaling within T cells also play an important role. Studies found impaired central Mitogen-activated protein kinase (MAPK) signaling associated with behavioral changes and cognitive deficits related to mood disorders, suggesting an important role of this cascade in psychiatric disorders [79]. MAPK proteins are involved in many cellular processes such as differentiation, proliferation, activation, and apoptosis, and may contribute to the immune alterations observed in BD [80]. Three major MAPK cascades are known, including the Extracellular signal-regulated protein kinase (ERK), C-Jun amino-terminal protein kinase/stress-activated protein kinase (JNK), and p38. During depressive episodes in BD patients, JNK levels in lymphocytes were lower than in euthymic patients and control individuals [81]. While phosphorylation of ERK1/2 is involved in cellular proliferation, differentiation, activation, and survival, phosphorylated p38 is related to cellular energy and pro-apoptotic fate, these two enzymes have reciprocal antagonistic actions [41]. BD is associated with increased percentage and phosphorylated protein levels of p-ERK in CD4^+^ and CD8^+^ T cells [41]. Increased p-ERK signaling may contribute in different ways to the immune/inflammatory imbalance observed in BD subjects. It is tempting to speculate that a lack of appropriate regulatory cells may allow the increase in p-ERK signaling in T-cell subsets, contributing to the immune imbalance (cell activation and pro-inflammatory profile). Another important intracellular signaling route related to the immune response is the nuclear factor kappa B (NF-κB) pathway. Alterations in the NF-κB pathway are associated with BD [82]. As a pleiotropic transcription factor, NF-κB is reported to mediate the effects of HPA axis dysregulation on stress-induced pleasure anhedonia and impaired neurogenesis [83], and NF-κB also regulates the balance between neuroprotective and pro-apoptotic mechanisms in the CNS via Nod-like receptor proteins 1/3 inflammasome regulation [84]. The NF-κB pathway is readily activated in response to different stimuli, and phosphorylation of the p65 subunit (p-p65) leads to its translocation to the nucleus to transcribe different pro-inflammatory genes. It has been shown that the percentage of p-p65^+^ T cells and the amount of p65 protein phosphorylation is increased in BD patients and that this phenomenon is closely related to the activation of T cells [40]. In conclusion, altered proportions of activated/regulatory lymphocyte subsets and differential intracellular signaling have been implicated in the immunological imbalance in BD.

#### Microbiome

The two-way communication between the gut microbiota and the brain is involved in neuronal development, brain function, cognitive regulation, and aging [85]. The decrease of *Bacteroides-Prevotella* group/*Enterobacter spp* in the gut suggested an imbalance in the intestinal microecology of BD patients, which was associated with illness severity and immune alterations, and a positive correlation between the *Enterobacter spp* count and the total T-cell ratio [86]. Multiple studies have documented an interaction between the gut microbiome, immunity, cognitive functioning, and behavior in several models, most of which involve rodents [87]. Th17 cells are only present in the lamina propria of the small intestine in healthy mice and are involved in the maintenance of the epithelial barrier by producing low amounts of IL17A [88]. Th17 cells are absent in germ-free mice, showing a major impact of the microbiota on Th17 cells [89]. Besides the microbial antigens, other signals are required to promote the differentiation of Th17 cells [90, 91]. For example, the trans-differentiation of Th17 cells to Tregs is partly dependent on the aryl hydrocarbon receptor, whose agonists are produced by the microbiota to promote anti-inflammatory responses consisting of tryptophan metabolites [92, 93]. Serum tryptophan metabolite levels were lower in bipolar individuals who were in a current state of euthymia or mild depression when compared to HC [94]. Tryptophan metabolites act as an intermediate in the inflammatory cascade to induce Tregs differentiation, limit Th1 and Th17 responses and produce anti-inflammatory mediators [95]. The presence of tryptophan depletion characteristic of mood disorders is a mechanism that explains the elevated Th17 and reduced Tregs present in BD patients [96, 97]. Microbial metabolites (e.g., short-chain fatty acids) also regulate Tregs homeostasis [98]. Polysaccharide A produced by *Bacteroides* species promotes Tregs’ anti-inflammatory effects while inhibiting Th1 and Th17 cell differentiation [99, 100]. Thus, by promoting Th17/Tregs imbalance, microbiota metabolites promote depression-like behaviors [101]. Other Th cells are associated with changes in the microbiota, such as helminth infections, which promote Th2 responses [102]. While animal models focus on the bacterial composition of the intestinal tract, studies to date in individuals with BD also point to the possible role of viruses. Human cytomegalovirus (HCMV), correlated with the clinical presentation of BD patients [103]. Under most circumstances, CMV infection is latent and asymptomatic, but it is associated with the expansion of late-differentiated CD8^+^ T cells, a reduced T-cell repertoire, reduced B-cell numbers, and increased IL-6 levels [104]. The HCMV infection may be a driving force in the process of early immune-senescence in BD [105]. In addition, CD8^+^ T cells play a principal role in the regulation of Epstein-Barr virus (EBV) infection and psychological stress compromises the ability of CD8^+^ T cells to prevent the reactivation of latent EBV infection [106, 107]. Stress may therefore increase the number of EBV-infected self-reactive B cells and plasma cells, thereby exacerbating BD, leading to increased stress and further affecting CD8^+^ T cell function in a vicious cycle [108]. At this stage, the pathways and potential mechanisms used by the microbiota to promote BD are only just emerging, all findings were associative, without establishing a causal relationship with BD pathogenesis, and their impact on T cell plasticity remains to be fully understood.

**Fig. 2:**
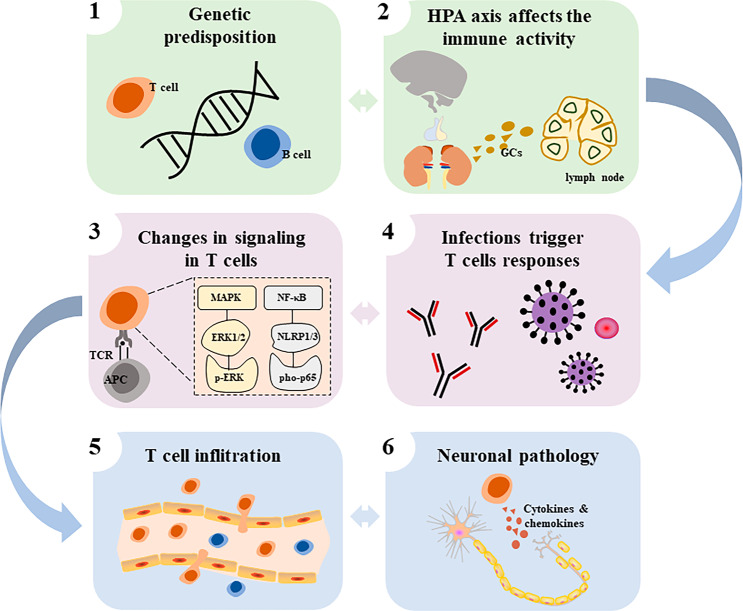
Potential mechanism(s) of altered T cell responses in BD. Possibly, (1) genetic variations change the adaptive immune composition and response in some individuals; (2) disruption of the HPA axis affects the immune response through the release of glucocorticoids; (3) with changes in inflammatory cascade signaling within T cells, there is an imbalance in the homeostasis of the intracellular environment; (4) in genetically susceptible individuals, (viral) infections may trigger cross-reactions of T cells to self-proteins associated with BD; (5) genetic susceptibility and BD pathology hampers brain-barrier-function, and increases its permeability; (6) within the brain parenchyma, T cells can change neuronal function and promote neuroinflammation. HPA Hypothalamic-pituitary-adrenal, TCR T cell receptor, APC antigen-presenting cell, MAPK mitogen-activated protein kinase, ERK extracellular signal-regulated protein kinase, NF-κB nuclear factor kappa B.

Overall, T cells are the point of convergence of many pathways associated with the stress response. The research investigating genetic variants linked to BD suggests that the genetic origins of immune imbalance may potentiate pathogen escape [109]. We propose a mechanism wherein genetic predisposition for BD contributes to an altered adaptive immune response in some individuals, and dysfunction of the HPA axis further exacerbates the immune response (Fig. 2). We postulate that in these individuals, the internal environment of T cells is altered, and (viral) infection more easily triggers a dysfunctional immune response. This process can generate and maintain neuroinflammation in BD, ultimately leading to the observed alterations in the proportion and function of T cell subsets. Whether the T cell response is protective or detrimental will likely depend on its nature. Potentially, increased levels of senescent and terminally differentiated T cells lead to hampered and dysfunctional immune responses, while a ‘young’ and flexible effector T cell response might alleviate early BD pathology. Different T cell subpopulations also correspond to different functions, and which signal(s) can be targeted to improve BD remains to be determined.

## Implications for patients with BD

### Increased comorbidities and infections

BD is now known to be associated not only with highly prevalent co-occurring psychiatric and substance use disorders but also with medical comorbidities, such as cardiovascular diseases, diabetes mellitus, obesity, and thyroid dysfunction. Repeatedly observed disorders of inflammation in BD, particularly defects in T-cell function, could explain some of the co-morbidities between BD and medical disorders. Excess mortality rates due to medical causes are between 1.5 and three times higher in adults with BD compared to the general population [110]. The increased mortality rate covers all natural causes of death, including cardiovascular, cerebrovascular, other medical disorders, and external causes of death. Cardiovascular mortality is the main cause of excess mortality in BD [110]. Inflammation plays a key role in cardiovascular disease, participating in atherosclerosis from its inception and development to its ultimate endpoint, thrombotic complications. It begins with inflammatory changes in the endothelium, which expresses the vascular cell adhesion molecule-1, which, in addition to attracting monocytes to transform into foam cells, also induces T cells to migrate to the endothelium and release pro-inflammatory cytokines, amplifying inflammatory activity and ultimately leading to atherosclerosis [111]. In symptomatic BD patients, lower BDNF was associated with greater mean carotid intima-media thickness, suggesting a potential interaction between neurotrophic/inflammatory biomarkers and atherosclerosis proxies as well as promising prevention strategies against cardiovascular disease in BD [112]. Activated oxidative and nitrosative stress pathways and a pro-atherogenic lipid profile have been found in both mood disorders and metabolic syndrome. Under these conditions, the levels of pro-inflammatory cytokines, and lipid peroxidation biomarkers, including malondialdehyde and atherogenic indices, are increased, as well as the levels of antioxidants [113]. These results suggest there may be a shared immune-inflammatory, oxidative and nitrosative stress, and metabolic pathways underpinning metabolic syndrome and BD. Another comorbid pathology with an inflammatory component is “autoimmune disease”, where people with BD are at increased risk of developing multiple sclerosis, psoriasis, inflammatory bowel disease, rheumatoid arthritis, and systemic lupus erythematosus [114]. A possible mechanistic overlap between mood disorders and autoimmune diseases has previously been proposed, with extravasation of activated CD8^+^ T cells in the peripheral circulation of BD patients reflecting T cell-mediated WM abnormalities in the brain parenchyma and a link between WM pathology and CD8^+^ T cell activation has been reported in other inflammatory diseases such as multiple sclerosis [65, 115]. Furthermore, as the classical role of CD8^+^ T cells is to mediate host defense against intracellular infection factors (especially viruses), a decrease in CD8^+^ T cells was observed even during remission, which could support the hypothesis of a close relationship between BD and infection caused by viruses [19]. Compared with the general population, BD patients are more predisposed to some infectious diseases, particularly hepatitis B or C and human immune-deficiency virus infection [116]. The increased incidence of certain viral diseases in BD patients may be the result of immune dysfunction, including T-cell defects, in the course of the disease, especially since studies have failed to demonstrate the presence of the viral genome in patients with psychiatric disorders [14]. Associations of BD with Borna disease virus, influenza virus, herpes simplex virus type 1, herpes simplex virus type 2, cytomegalovirus, human herpes virus 6, and Toxoplasma gondii suggest that these relationships may not be specific to any one pathogen but rather involve a common mechanism, possibly immune activation [109]. These suggest that alterations in the immune system, particularly in T-cell function in BD patients, are associated with negative outcomes in the long term.

### Potential pharmacological outcomes

Reciprocally, it also raises the question of how to mitigate T-cell defects and thus treat BD. The classical mood stabilizers (lithium, valproic acid) have been shown to directly affect the proliferation and activity of T cells, both of which protect T lymphocytes from apoptosis, and the inhibition of T lymphocyte proliferation is visible only at toxic doses [117]. In addition to down-regulating IL-6 levels in BD manic phase, lithium, and valproic acid also affect inflammatory pathways by down-regulating T-cell activation markers IL-2 and IFN-γ and reducing lipopolysaccharide-induced dopaminergic neurotoxicity by inhibiting microglial overactivation [118, 119]. Lithium also improves depressed mood in BD and has a broader effect on T cells than valproic acid, which is primarily used to treat manic episodes or for prophylaxis. Lithium enhances lymphocyte activity by increasing lymphocyte responses to antigens and mitogens, promoting immunoglobulin synthesis or enhancing natural killer activity, and enhancing BDNF mRNA levels in lymphocytes from BD patients [120]. Lithium also affects specific T-cell phenotypes. In BD patients treated with Li, the number of CD4^+^T cells decreased, while the number of CD8^+^T cells increased, and the ratio of CD4^+^T/CD8^+^T was negatively correlated with the duration of lithium treatment [121]. In colonic diseases, lithium alleviated inflammatory responses in a G-protein coupled receptor 43-dependent manner by activating Tregs responses [122]. In a mouse model of autoimmune encephalomyelitis, lithium attenuated IFN-γ-dependent activation of naïve T cells and transcription factor STAT1 in inflammatory T cells and Th1 cells [123]. Lithium acts through multiple pathways to inhibit Glycogen synthetase kinase-3β. This enzyme phosphorylates and inhibits nuclear factors that turn on cell growth and protection programs, including the nuclear factor of activated T cells and WNT/β-catenin [120]. The immunomodulatory effects of mood stabilizers are beginning to emerge, but the efficacy of immunomodulatory/anti-inflammatory therapy for BD remains controversial in clinical studies. It has been suggested that anti-inflammatory drugs might confer a moderate antidepressant effect in bipolar depression [124, 125]. For example, TNF-α inhibitors (adalimumab, infliximab, etanercept, and certolizumab) have been shown to reduce depressed mood, inhibit Th cell proliferation and function and improve cognitive decline in BD [126]. Minocycline has anti-depressive effects in bipolar depression, probably by decreasing the pro-inflammatory cytokine levels [127]. Husain et al. questioned the potential therapeutic benefit of adjuvant anti-inflammatory drugs for the acute treatment of BD, and they have found no evidence that minocycline or celecoxib is superior to placebo in the treatment of BD [128]. McIntyre et al. found similar results, with infliximab not significantly reducing depressive symptoms compared to placebo in adults with bipolar depression [45]. Interestingly, they reported a significant reduction in depressive symptoms in subgroups of physical and/or sexual abuse following infliximab treatment [45]. Disparate anti-inflammatory drugs have variable antidepressant effects in adults with unipolar and bipolar depressive disorders [125, 129]. These variable effects suggest that select anti-inflammatory agents may be differentially efficacious in a subset of individuals with bipolar disorder who are exhibiting proinflammatory balance. The relatively high rate of negative and failed trials in bipolar depression, as well as the variable response to existing treatments, implicates distinct biotypes within heterogeneous populations with bipolar disorders [45, 130]. Further studies are needed to confirm whether there is a plausible causal relationship between abnormal inflammatory processes including T-cell defects and BD disease activity so that further therapeutic trials of adjuvant anti-inflammatory agents for BD can be targeted. In addition, light therapy indirectly promotes cytokine production in Th1 cells and reduces the anti-inflammatory response of Th2 cells by enhancing melatonin on the one hand, and regulates lymphocyte function by directly restoring the balance between Th1 and Th2 functions and increasing the release of IL-2 and IL-10 [131, 132]. The therapeutic role of EBV-specific T-cell therapy in BD has also been reported in the literature, considering the role of EBV infection in the pathogenesis of BD [108]. Many potential new strategies for normalizing neuroimmune pathways require further validation for their promise in BD treatment, and a better understanding of the role of the immune system, including T cells, in BD is a prerequisite for designing the most effective drugs.

## Conclusion

**Fig. 3:**
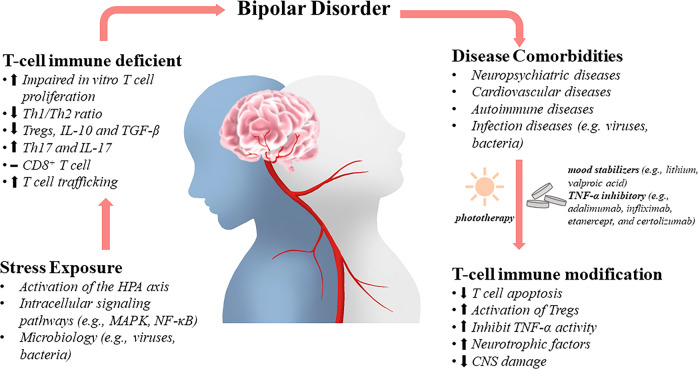
Brain-T cell interactions in BD. Stress exposure leads to the activation of neuroendocrine and inflammatory pathways, and infection further exacerbates T-cell dysfunction, as evidenced by altered T-cell subpopulation ratios, impaired proliferation in response to non-specific and specific stimuli, and increased infiltration of the brain parenchyma. This T-cell dysfunction may in turn promote disease progression. Drugs and other targeted T-cell therapies exhibit neuroprotective and anti-inflammatory properties. Modified T-cell activities may also subserve resilience against stressors and maintain neuronal integrity during health and illness.

Immunoregulation is inherently intricate and complicated, and when taken together with diversified pathological, or physiological factors, the precise manipulation of T lymphocytes to achieve better intervention, therapeutics, or vaccination seems even more difficult (Fig. 3). Animal studies that have addressed underlying pathological mechanisms emphasize the value of targeting peripheral immunity to combat BD pathology. Due to numerous obstacles to BD-related research, including the lack of exact animal models, the difficult accessibility to pathological brain samples from patients, the timing of sampling (mania, depression, relapse and remission in BD, medication selection), the size of clinical samples, as well as the understanding of the so-called inherent etiologies, the roles of T lymphocytes in BD are merely based on observations and evaluations. As the boundaries of neuroinflammation expand, investigations on the intercorrelated or even shared functions of Th subsets in different disease settings could promote a deeper understanding of this complex immune network. In turn, through comparing distinct contributions of Th subsets under different neuroinflammatory conditions, more precise comprehension of pathogenesis, prevention, therapeutics, and prognosis of these diseases may be achieved. Comparing distinct contributions of T-cell subsets allows for more precise comprehension of BD pathogenesis, prevention, therapeutics, and prognosis.

It is worth noting that the exact nature of the relationship between T cell imbalance and BD is still unclear, whether T cell imbalance is the main cause of the pathological process of BD, a consequence of the disease, or just a bystander effect, more studies are needed to elucidate its correlation with the disease and the mechanism of changes in different T cell subsets. For example, the involvement of T cells in the preclinical stages of BD could be further investigated using longitudinal studies that follow healthy individuals over time. Eventually, understanding the function of T cells and their migration to the CNS and further mapping the targeted antigens of “BD-specific T cells” will provide us with specific windows and targets in time and space, which can potentially be used to develop intervention and modulation strategies in BD.

## Supplementary information

Supplement Figure 1-Description

Quality assessment of the included studies.

A search strategy was developed based on the research question, according to the PICO format.
