# Supplementary material for: T cells: an emerging cast of roles in bipolar disorder
Source: Transl Psychiatry. 2023 May 8;13:153. doi: 10.1038/s41398-023-02445-y (PMC10167236; doi:10.1038/s41398-023-02445-y)
Supplement: Supplementary file 1 — Supplement Figure 1-Description [file 41398_2023_2445_MOESM1_ESM.docx]

**A search strategy was developed based on the research question, according to the PICO format.**

Studies were identified using the following databases: PubMed, Embase, and Web of Science from any time to February 19th 2023. The systematic search was performed by two independent researchers blinded to each other’s results (Zhenni Chen, Yiran Huang).

The following search strategy was used in PubMed and then adapted for Embase and Web of Science: (bipolar OR manic OR mania) AND (T lymphocyte subsets OR CD3+T OR CD4+T OR T helper OR CD8+T OR cytotoxic T OR Tregs), no restrictions were applied. The search yielded 322 articles: (PubMed = 92, Embase = 79, Web of Science = 151).

To determine whether an article was relevant to our study, we used the following inclusion criteria: the study should (1) present original data, and (2) assessment of T lymphocyte subsets changes in BD patients and HC. The exclusion criteria were: (1) animal, in vitro, and cell culture studies, (2) reviews and meta-analyses, and (3) case reports.

The studies were selected by two blinded reviewers (Zhenni Chen and Yiran Huang) who determined if the studies met the inclusion criteria. Manuscripts were assessed independently by the two raters and divergences were resolved by consensus in a meeting with another researcher (Bingqi Wang). Firstly, the raters screened articles by title and abstract, and after by full text. Duplicates, review articles, and articles not fulfilling the search criteria were removed. The details of the search strategy are depicted in Supplement Figure 1.
