# Supplementary material for: T cells: an emerging cast of roles in bipolar disorder
Source: Transl Psychiatry. 2023 May 8;13:153. doi: 10.1038/s41398-023-02445-y (PMC10167236; doi:10.1038/s41398-023-02445-y)
Supplement: Supplementary file 2 — Quality assessment of the included studies. [file 41398_2023_2445_MOESM2_ESM.docx]

| Study | Selection | | | | Comparability  Comparability of cases and controls on the basis of the design or analysis | Exposure | | | Scores |
| --- | --- | --- | --- | --- | --- | --- | --- | --- | --- |
|  | Is the case definition adequate | Representativeness of the cases | Selection of Controls | Definition of Controls |  | Ascertainment of exposure | Same method of ascertainment for cases and controls | Non-Response rate |  |
| Wahlin A et al., 1984 [1] | * | * | * |  | * | * | * |  | 6 |
| Wilson R et al., 1991[2] | * | * |  |  | * | * | * |  | 5 |
| Breunis MN et al., 2003 [3] | * | * | * | * | ** | * | * | * | 9 |
| Torres KC et al., 2008 [4] | * |  | * |  | * | * | * |  | 5 |
| Drexhage RC et al., 2011 [5] | * | * | * | * | * | * | * | * | 8 |
| Wieck A et al., 2013 [6] | * | * | * | * | ** | * | * | * | 9 |
| do Prado CH et al., 2013 [7] | * |  |  | * | * | * |  |  | 4 |
| Barbosa IG et al., 2014 [8] | * | * | * |  | * | * | * | * | 7 |
| Wu W et al., 2017 [9] | * | * | * |  | * |  | * | * | 6 |
| Vogels RJ et al., 2017 [10] | * | * | * | * | * |  | * | * | 7 |
| Poletti S et al., 2017 [11] | * | * | * | * | * | * | * | * | 8 |
| Becking K et al., 2018 [12] | * |  | * |  | * | * | * |  | 5 |
| Magioncalda P et al. 2018 [13] | * | * | * | * | * | * | * | * | 8 |
| Counotte J et al., 2018 [14] | * | * | * | * | ** | * | * | * | 9 |
| Snijders G et al., 2019 [15] | * | * | * |  | * | * | * |  | 6 |
| Lu Q et al., 2019 [16] | * |  | * |  | * | * | * |  | 5 |
| Wu TN et al., 2019 [17] | * | * | * |  | * | * | * |  | 6 |
| Pietruczuk K et al., 2019 [18] | * | * | * |  | * | * | * |  | 6 |
| Maes M et al., 2021 [19] | * | * | * |  | * | * | * | * | 7 |
| Su L et al., 2022 [20] | * |  | * |  | * | * | * |  | 5 |

**Supplement Table 1** Quality assessment of the included studies.

**Reference:**

1. Wahlin A, von Knorring L, Roos G. Altered distribution of T lymphocyte subsets in lithium-treated patients. Neuropsychobiology. 1984;11:243-46.
2. Wilson R, McKillop JH, Crocket GT, Pearson C, Jenkins C, Burns F, et al. The effect of lithium therapy on parameters thought to be involved in the development of autoimmune thyroid disease. Clin Endocrinol (Oxf). 1991;34:357-61.
3. Breunis MN, Kupka RW, Nolen WA, Suppes T, Denicoff KD, Leverich GS, et al. High numbers of circulating activated T cells and raised levels of serum IL-2 receptor in bipolar disorder. Biol Psychiatry. 2003;53:157-65.
4. Torres KC, Souza BR, Miranda DM, Nicolato R, Neves FS, Barros AG, et al. The leukocytes expressing DARPP-32 are reduced in patients with schizophrenia and bipolar disorder. Prog Neuropsychopharmacol Biol Psychiatry. 2009;33:214-9.
5. Drexhage RC, Hoogenboezem TH, Versnel MA, Berghout A, Nolen WA, Drexhage HA. The activation of monocyte and T cell networks in patients with bipolar disorder. Brain Behav Immun. 2011;25:1206-13.
6. Wieck A, Grassi-Oliveira R, do Prado CH, Rizzo LB, de Oliveira AS, Kommers-Molina J, et al. Differential neuroendocrine and immune responses to acute psychosocial stress in women with type 1 bipolar disorder. Brain Behav Immun. 2013;34:47-55.
7. do Prado CH, Rizzo LB, Wieck A, Lopes RP, Teixeira AL, Grassi-Oliveira R, et al. Reduced regulatory T cells are associated with higher levels of Th1/TH17 cytokines and activated MAPK in type 1 bipolar disorder. Psychoneuroendocrinology. 2013;38:667-76.
8. Barbosa IG, Rocha NP, Assis F, Vieira É L, Soares JC, Bauer ME, et al. Monocyte and lymphocyte activation in bipolar disorder: a new piece in the puzzle of immune dysfunction in mood disorders. Int J Neuropsychopharmacol. 2014;18:pyu021.
9. Wu W, Zheng YL, Tian LP, Lai JB, Hu CC, Zhang P, et al. Circulating T lymphocyte subsets, cytokines, and immune checkpoint inhibitors in patients with bipolar II or major depression: a preliminary study. Sci Rep. 2017;7:40530.
10. Vogels RJ, Koenders MA, van Rossum EF, Spijker AT, Drexhage HA. T Cell Deficits and Overexpression of Hepatocyte Growth Factor in Anti-inflammatory Circulating Monocytes of Middle-Aged Patients with Bipolar Disorder Characterized by a High Prevalence of the Metabolic Syndrome. Front Psychiatry. 2017;8:34.
11. Poletti S, de Wit H, Mazza E, Wijkhuijs AJM, Locatelli C, Aggio V, et al. Th17 cells correlate positively to the structural and functional integrity of the brain in bipolar depression and healthy controls. Brain Behav Immun. 2017;61:317-25.
12. Becking K, Haarman BCM, Grosse L, Nolen WA, Claes S, Arolt V, et al. The circulating levels of CD4^+^ t helper cells are higher in bipolar disorder as compared to major depressive disorder. J Neuroimmunol. 2018;319:28-36.
13. Magioncalda P, Martino M, Tardito S, Sterlini B, Conio B, Marozzi V, et al. White matter microstructure alterations correlate with terminally differentiated CD8^+^ effector T cell depletion in the peripheral blood in mania: Combined DTI and immunological investigation in the different phases of bipolar disorder. Brain Behav Immun. 2018;73:192-204.
14. Counotte J, Drexhage HA, Wijkhuijs JM, Pot-Kolder R, Bergink V, Hoek HW, et al. Th17/T regulator cell balance and NK cell numbers in relation to psychosis liability and social stress reactivity. Brain Behav Immun. 2018;69:408-17.
15. Snijders G, Brouwer R, Kemner S, Bootsman F, Drexhage HA, Hillegers MHJ. Genetic and environmental influences on circulating NK and T cells and their relation to bipolar disorder. Int J Bipolar Disord. 2019;7:4.
16. Lu Q, Lai J, Lu H, Ng C, Huang T, Zhang H, et al. Gut Microbiota in Bipolar Depression and Its Relationship to Brain Function: An Advanced Exploration. Front Psychiatry. 2019;10:784.
17. Wu TN, Lee CS, Wu BJ, Sun HJ, Chang CH, Chen CY, et al. Immunophenotypes associated with bipolar disorder and lithium treatment. Sci Rep. 2019;9:17453.
18. Pietruczuk K, Lisowska KA, Grabowski K, Landowski J, Cubała WJ, Witkowski JM. Peripheral blood lymphocyte subpopulations in patients with bipolar disorder type II. Sci Rep. 2019;9:5869.
19. Maes M, Nani JV, Noto C, Rizzo L, Hayashi MAF, Brietzke E. Impairments in Peripheral Blood T Effector and T Regulatory Lymphocytes in Bipolar Disorder Are Associated with Staging of Illness and Anti-cytomegalovirus IgG Levels. Mol Neurobiol. 2021;58:229-42.
20. Su L, Shuai Y, Mou S, Shen Y, Shen X, Shen Z, et al. Development and validation of a nomogram based on lymphocyte subsets to distinguish bipolar depression from major depressive disorder. Front Psychiatry. 2022;13:1017888.
