# Supplementary figures and images for: T cells: an emerging cast of roles in bipolar disorder
Source: Transl Psychiatry. 2023 May 8;13:153. doi: 10.1038/s41398-023-02445-y (PMC10167236; doi:10.1038/s41398-023-02445-y)

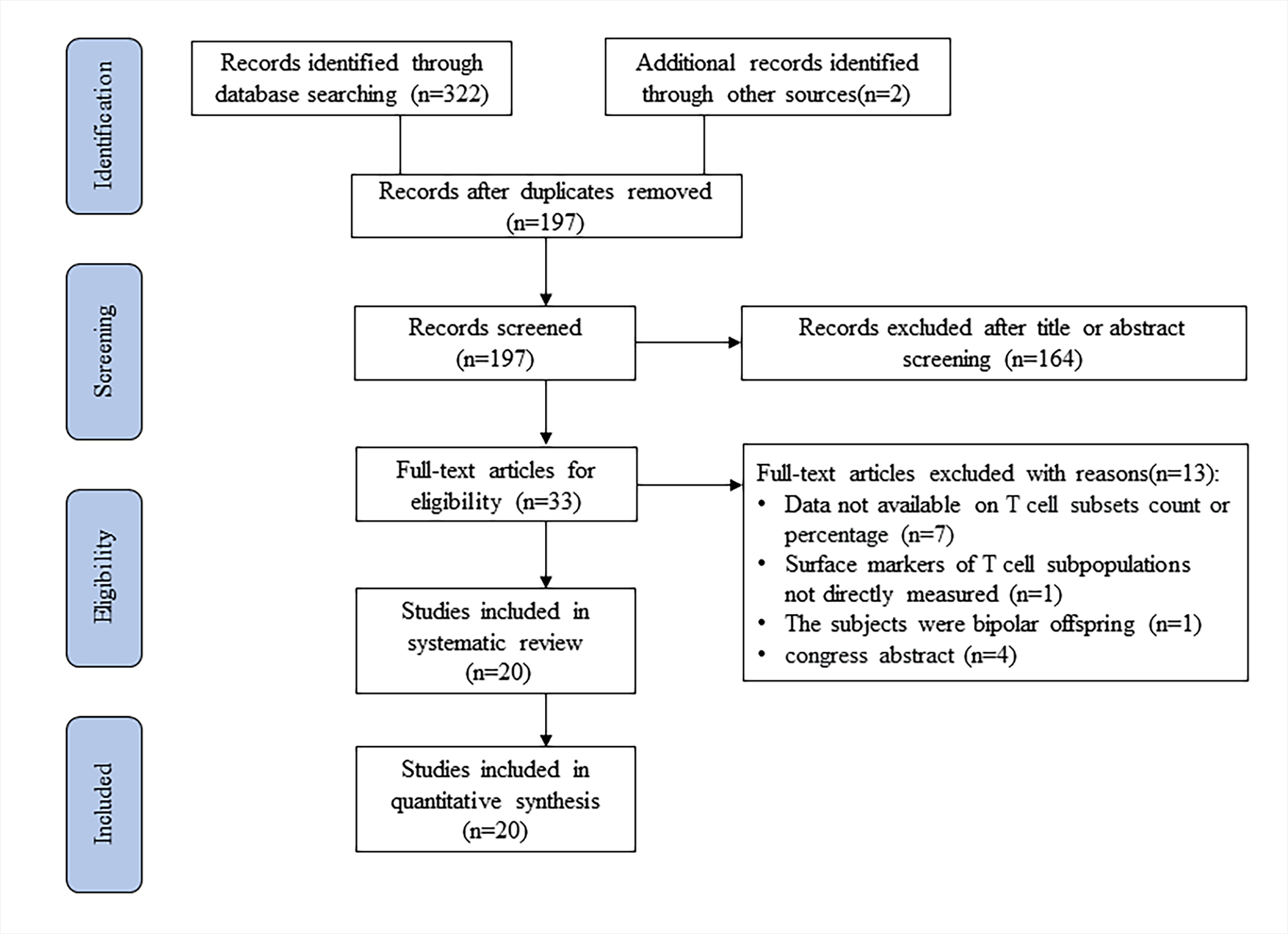

Supplement: Supplementary file 3 — A search strategy was developed based on the research question, according to the PICO format. [file 41398_2023_2445_MOESM3_ESM.tif]
